# Overvalued Ideas: Conceptual Analysis and Literature Review

**DOI:** 10.3390/bs16050708

**Published:** 2026-05-05

**Authors:** Jennifer Dork, Eugene Dimenstein, Lawrence Burns, Megan Demshuk

**Affiliations:** Department of Psychology, Grand Valley State University, Allendale, MI 49401, USA; dimenste@mail.gvsu.edu (E.D.); demshukm@mail.gvsu.edu (M.D.)

**Keywords:** overvalued ideas, delusion, obsession, insight, ego-syntonicity

## Abstract

The concept of the overvalued idea (OVI) has been debated since German psychiatrist Karl Wernicke coined the term in the late nineteenth century, describing it as an emotionally exaggerated yet psychologically comprehensible belief arising from normal cognitive processes that becomes central to an individual’s mental life. Since that time, the construct has been variably reinterpreted through competing theoretical lenses, ranging from Kraepelin’s biological nosology to contemporary cognitive-behavioral and multidimensional models. Despite its clinical relevance across disorders (e.g., anorexia nervosa, body dysmorphic disorder, obsessive–compulsive disorder), overvalued ideas remain inconsistently and insufficiently defined, having been alternately treated as attenuated delusions, markers of poor insight, or disorder-specific severity indicators—interpretations that have limited theoretical coherence, measurement precision, and clinical utility. This review traces the historical evolution of the overvalued idea, clarifies enduring theoretical misinterpretations, and proposes a comprehensive, practical definition. Integrating historical and contemporary perspectives, we define an overvalued idea as a psychologically intelligible, ego-syntonic belief held with disproportionate emotional significance that dominates cognition and behavior without meeting criteria for an obsession or a delusion. We further propose that overvalued ideation is best conceptualized as a transdiagnostic mechanism through which emotionally reinforced beliefs acquire pathological dominance across disorders, a formulation that both honors Wernicke’s original insight and can be operationalized for future research, measurement, diagnosis, and treatment.

## 1. Introduction

The term overvalued idea (OVI) is commonly used to describe a false, exaggerated, and motivationally significant belief held less intensely than a delusion (American Psychiatric Association, 2022). Despite its rich historical significance, the concept has received comparatively limited systematic conceptual integration in contemporary research, motivating the present narrative review and theoretical synthesis. Across their development, overvalued ideas have been variably conceptualized—as attenuated delusions, markers of poor insight, or emotionally inflated beliefs—within inconsistent frameworks (Kristinsdottir et al., 2025; Veale, 2002). The lack of consensus has limited theoretical coherence, measurement precision, and clinical utility.

Despite its enduring relevance, the construct remains inconsistently defined in both research and practice. Diagnostic systems often rely on incomplete, vague, or reductive descriptions. For example, the Diagnostic and Statistical Manual of Mental Disorders does not provide a formal definition of overvalued ideas, instead referencing them indirectly within insight specifiers and disorder-specific descriptions, which may limit conceptual clarity and consistency in their identification (Diagnostic and Statistical Manual of Mental Disorders, Fifth Edition, Text Revision; American Psychiatric Association, 2022). Separately, the American Psychological Association (APA) defines OVIs as “false or exaggerated beliefs that are maintained by an individual (e.g., the idea that one is indispensable in an organization) (American Psychological Association, n.d.). They are described as less rigidly and persistently held than delusions, with an implied nonconscious motivation that, if made conscious, would reduce their importance and corresponding dysfunction. Existing conceptualizations have often emphasized discrete features, such as insight, conviction, or belief content—without fully specifying how these components cohere within a broader cognitive–affective–behavioral system (Kristinsdottir et al., 2025).

To address this limitation, the present review advocates for a multidimensional, process-based model of overvalued ideation. Within this framework, OVIs are not defined along a single continuum or solely by discrete features contrasted with related constructs (i.e., delusions and obsessions). Instead, they are conceptualized as emerging from the interaction of multiple domains (i.e., belief conviction, emotional intensity, insight variability, cognitive rigidity, social validation, behavioral salience and reinforcement, etc.), rather than any single defining feature. This approach emphasizes a mechanism, rather than static classification, through which emotionally charged, reinforced beliefs acquire and maintain disproportionate influence over cognition and behavior.

We propose that overvalued ideation is best understood as a transdiagnostic mechanism that operates across a range of psychological disorders and, in some cases, independently of formal diagnostic categories, rather than being confined to any single disorder (Wade et al., 2025). The ideas and beliefs that manifest from this process may take on various thematic forms (e.g., morality, health, appearance) but share underlying mechanisms of development and maintenance (e.g., emotional amplification, reinforcement, cognitive rigidity). This perspective emphasizes a process-based multidimensional model that accounts for individual differences between multiple interactive continua (Hofmann & Hayes, 2019; Ong et al., 2022).

## 2. Methods

Establishing a comprehensive and operationalized conceptualization has important implications for both research and clinical practice. For researchers, a refined framework provides a foundation for systematically investigating OVIs as a distinct construct and developing valid and reliable measurement tools. A clear and consistent definition can help clinicians identify key cognitive and behavioral markers of OVI—such as emotional amplification, value-based belief dominance, and resistance to change—that may otherwise be misclassified or overlooked and can ultimately improve treatment precision and outcomes. Wade et al. (2025) demonstrate that identifying transdiagnostic processes can improve early mental health intervention in youth. Recognizing the presence and salience of overvalued ideation may enable clinicians to optimize treatment by targeting mechanisms of belief dominance and tailoring interventions to features that distinguish OVIs from obsessions and delusions.

This review adopts a narrative and conceptual approach rather than a systematic or scoping methodology. A narrative literature review is a selective, theory-driven synthesis of research that aims to integrate findings, identify patterns, and develop new conceptual frameworks rather than systematically aggregate all available evidence (Baumeister & Leary, 1995; Theile & Beall, 2024). Sources were selected to represent historically foundational texts, influential theoretical formulations, and clinically salient models that have shaped the understanding of overvalued ideas across psychiatry, psychology, and related fields. Emphasis was placed on seminal contributions, widely cited frameworks, and representative contemporary perspectives rather than an exhaustive enumeration of all available studies. The primary aim of this review is conceptual clarification and theoretical integration, not quantitative synthesis or formal evidence weighting. Accordingly, the review prioritizes coherence, interpretive transparency, and critical synthesis to illuminate enduring ambiguities and guide future research. Because the aim of this review is conceptual clarification, historically foundational and seminal sources are retained where they most directly articulate original theoretical positions; recent literature is incorporated to contextualize and extend these contributions, but does not supersede primary sources when the claim pertains to their original formulations.

## 3. Historical Foundations of the Overvalued Idea

This section outlines the historical development of the term overvalued idea, beginning with its initial formulation in late nineteenth-century psychiatry and tracing subsequent theoretical challenges and refinements.

### 3.1. Karl Wernicke’s Original Formulation

Wernicke (1848–1905) coined the term overvalued idea in 1892, during a time when psychology was still in its infancy and characterized by competing paradigms. In nineteenth-century Germany, the dominant approach was clinical neuropsychiatry, which aimed to describe mental phenomena in terms of brain structure and function (Kendler, 2022; Gelder, 1976). This perspective contrasted with introspective psychology, which sought to understand mental life through subjective experience. Wernicke, trained under Meynert, was part of the neuropsychiatric movement, which viewed psychological disorders as arising from disruptions in neural functioning and aimed to understand them through the observation of anatomical and physiological mechanisms.

Before developing the concept of the overvalued idea, Wernicke had already established himself through work on neural circuits, sensory processing, and language comprehension, as well as the identification of Wernicke’s aphasia (S. Ahmad, 2024). When he became director of the Breslau Psychiatric Clinic, Wernicke’s focus expanded from neuroanatomy to psychopathology. Here, his perspective became more nuanced, shifting from reductionism to viewing the psychological experiences he observed in his patients as understandable disturbances in normal functioning (S. Ahmad, 2024). He approached these experiences through the lens of associationism and the interplay of multiple psychological processes, employing a data-driven approach rooted in neurosurgery and neurohistology (S. Ahmad, 2024; Berrios, 1996).

One key challenge in his clinical descriptions was observing patients whose dominant, emotionally charged beliefs significantly influenced their behavior without meeting the criteria for delusion. Wernicke sought to explain these “gray areas” beyond an anatomical perspective. These beliefs were exaggerated, persistent, and behaviorally significant, yet they were understandable within the context of the individual’s personality and circumstances. To address this conceptual gap, Wernicke coined the term overvalued idea (Wernicke, 1900; Veale, 2002; Kristinsdottir et al., 2025).

Wernicke’s descriptions have been interpreted as suggesting that overvalued ideas arise from normal associative processes that become pathologically intensified through emotional amplification, rather than due to processes of psychosis or lesion (Berrios, 1996; Crisan et al., 2022). Drawing on his earlier models of associative neural networks, he suggested that emotionality could lead an ordinary idea to dominate a person’s mental life and behaviors. Subsequent theorists linked Wernicke’s conceptualization to several disorders, including anorexia nervosa, body dysmorphic disorder, morbid jealousy, parasitophobia, hypochondriasis, and quarrelsome paranoid state (Barton et al., 2022; P. J. McKenna, 1984; Rahman et al., 2020). While many of these diagnoses did not exist during his time, they are in line with what he described in Grundriss der Psychiatrie in Klinischen Vorlesungen, in the year 1900 (Barton et al., 2022; Kristinsdottir et al., 2025; P. J. McKenna, 1984; Rahman et al., 2020; Wernicke, 1900). He also suggested that overvalued ideas were associated with poor prognosis and resistance to treatment. His work introduced the overvalued idea as a psychologically understandable exaggeration and overemphasis of normal thought or belief, beginning the process of distinguishing it from the incomprehensible and detached delusion.

### 3.2. Emil Kraepelin’s Nosological Reinterpretation

Kraepelin (1856–1926), a contemporary and occasional intellectual rival of Wernicke, expanded upon and reinterpreted his colleague’s ideas through a distinctly biological and nosological framework (Ebert & Bär, 2010; Heckers, 2020; Hoff, 2015; Huda, 2023). Trained in medicine and psychiatry and heavily influenced by Wilhelm Wundt, Kraepelin is often regarded as the founder of modern psychiatric nosology, the classification system that inspired contemporary diagnostic systems (e.g., DSM and ICD) (Hoff, 2015; Huda, 2023). His goal was to analyze and describe psychological disorders, particularly psychosis, based on their recorded onset, course, and outcomes, rather than solely on a set of symptoms specific to each disorder (Heckers, 2020; Hoff, 2015; Huda, 2023; Jaspers, 1913/1963). He aimed to classify disorders less by pure symptomatology and more by patterns and trajectories, placing greater emphasis on observability (Ebert & Bär, 2010; Huda, 2023). At a time when psychiatry was fragmented between neuroanatomical research and speculative introspection, Kraepelin focused on organized diagnostics based on predictable trajectories.

Within this emerging medicalized model, Kraepelin reinterpreted many of Wernicke’s psychologically nuanced concepts, including the notion of overvalued ideas, although he did not use the term directly (Kendler, 2025; Heatherton & Baumeister, 1991; P. J. McKenna, 1984). Contrary to Wernicke, Kraepelin rejected the notion that such phenomena reflected exaggerated normal functioning; instead, he conceptualized them as expressions of an underlying pathological process and classified similar presentations within paranoid illness categories. He occasionally referred to these presentations as a ‘secondary form’ of paranoia, positioning them within a pathological trajectory of delusion progression (Kendler, 2025; P. J. McKenna, 1984). Kraepelin later revised his stance, acknowledging that certain extreme beliefs could be attributed to external events and even recognizing the significance of emotion (Marcuse, 1913; P. J. McKenna, 1984). His influence reshaped the trajectory of psychiatry, as his commitment to a medical model emphasizing symptom clusters, disease course, and prognosis encouraged increasingly biological interpretations of mental disorder. In this paradigm, the concept of overvalued ideas lost much of its psychological nuance and theoretical explanatory depth. Despite this narrowing, Kraepelin’s reclassification laid the groundwork for modern diagnostic frameworks (Heckers et al., 2022; Huda, 2023; Insel et al., 2022). While speculative, it is plausible that Wernicke would have taken an initiative to oppose Kraepelin’s inclination to categorize these phenomena as paranoid or delusional diseases. Unfortunately, Wernicke’s early death in 1905 prevented him from addressing the growing influence of Kraepelinian nosology (S. Ahmad, 2024; Goedert et al., 2007; Hoy et al., 2023). This nosological reframing raised a conceptual dilemma that Jaspers later addressed: Should the distinction between overvalued ideas and delusions be based on prognosis and disease classification, as suggested by Kraepelin, or on phenomenological understandability and meaning, as proposed by Wernicke?

### 3.3. Karl Jaspers’ Phenomenological Mediation

In response to the emerging divide, Jaspers (1883–1969) revisited the conceptual dispute between Wernicke and Kraepelin, offering a mediating framework that clarified the conceptual boundary between their perspectives. Trained in medicine before turning to philosophy, Jaspers contributed a humanistic outlook to psychiatry (Ikkos et al., 2025). He emphasized the importance of understanding patients’ subjective experience rather than relying solely on objective observation or biological explanations, and endorsed the existence of an OVI, aligning more closely with Wernicke (Jablensky, 2013; P. J. McKenna, 1984; Spencer & Broome, 2023). However, he disagreed with Wernicke’s associative approach, suggesting that Wernicke was engaging in “brain mythology” by making diagnostic generalizations based on associations between localized brain regions (García-Molina & Peña-Casanova, 2022, p. 180). Jaspers distinguished overvalued ideas (OVIs) from delusions based on their understandability. He argued that an overvalued idea arises from normal psychological processes and is meaningfully linked to an individual’s experiences, values, and emotions; “Mood-states, wishes and drives give rise to delusion-like ideas (overvalued ideas) which arise in more or less understandable fashion from them” (Jaspers, 1913/1959). In contrast, a delusion is fundamentally “un-understandable,” emerging without a psychologically meaningful context (Mishara & Fusar-Poli, 2013, p. 178; Wendler & Fuchs, 2023). He rejected the notion that OVIs are merely less intense delusions (Jablensky, 2013; Jaspers, 1913/1959; Mishara & Fusar-Poli, 2013; Wendler & Fuchs, 2023). Jaspers’ contribution offered a conceptual boundary that distinguished phenomenological understanding from nosological explanation. Through Jaspers, the overvalued idea was conceptualized as a meaningful, ego-syntonic phenomenon capable of causing significant distress and behavioral consequences.

### 3.4. Aubrey Lewis’s Practical Description

Building on Jasper’s distinctions, Lewis (1900–1975) provided one of the earliest English-language descriptions of the OVI. Lewis was a prominent figure in British psychiatry and emphasized rigorous descriptions, clinical observation, and practical applications, reflecting early forms of descriptive and social psychology. His orientation is reflected in his analysis of belief pathology, seeking to operationalize the overvalued idea for psychiatric assessment by delineating observable components that distinguished OVIs from delusions and obsessions (Barton et al., 2022; Gelder, 1976; Kraepelin, 1905; Lewis, 1936; P. McKenna, 2017; Mishara & Fusar-Poli, 2013). Consistent with Wernicke, he presented OVIs, or ideas that resemble OVIs, through a modern lens, as emotionally charged ideas distinct from delusions, and not merely early delusional patterns of thought (Barton et al., 2022; Kraepelin, 1905; Lewis, 1936; P. McKenna, 2017). Congruent with Jaspers’ framework, he emphasized their understandability and plausibility in the context of personality as a distinguishing factor between idea types. While he did not propose a mechanistic model or framework, he further solidified the concept of an overvalued idea in the context of descriptive and practical settings. He wrote in extensive detail about various forms of ideas that he observed among his patients, carefully delineating different features of such ideas and the various patterns he observed. In his writings, Lewis carefully documented variations in how ideas functioned across patients, attending to features such as emotional investment, consistency with personality, degree of resistance, and behavioral significance (Lewis, 1936). Although the term overvalued idea was not explicitly used, Lewis consistently differentiated belief phenomena across multiple clinical accounts by attending to whether ideas were experienced as obsessional or belief-like, acknowledged as irrational, approached delusional conviction, aligned with personality, and exerted disproportionate emotional or behavioral influence (Lewis, 1936, pp. 325–336). His work further paved the way for OVIs to be considered and applied as a distinct theoretical and clinical concept.

**Figure 1:**
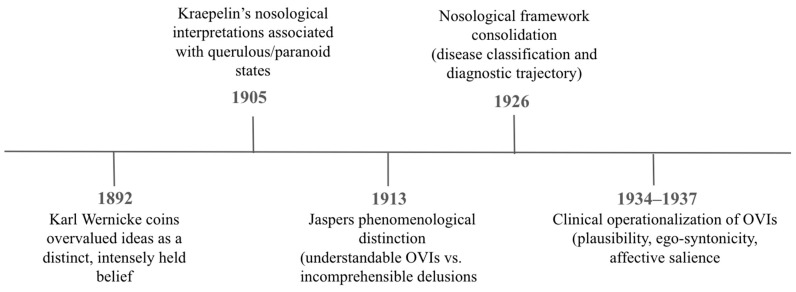
Historical development of the concept of overvalued ideas, from early phenomenological descriptions to later nosological and conceptual distinctions (Berrios, 1996; Ebert & Bär, 2010; Heckers, 2020; Hoff, 2015; Jablensky, 2013; Jaspers, 1913/1959, 1913/1963; Kendler, 2018; Kraepelin, 1905; Lewis, 1936; P. J. McKenna, 1984; Veale, 2002; Wernicke, 1900) Note. Figure created by the authors.

As displayed in Figure 1, Wernicke, Kraepelin, Jaspers, and Lewis collectively form the foundational quartet in the historical development of the concept of the overvalued idea. Wernicke’s affective and associative model laid the groundwork for understanding how ego-syntonic and culturally shared ideas can become inflated and maladaptive in both function and psychology (Crisan et al., 2022). Kraepelin’s biological framework shifted the focus toward disease classification, emphasizing progression and outcomes, and Jaspers served as a theoretical mediator, emphasizing understandability. Lewis helped operationalize and apply the concept of OVIs in clinical contexts, contributing to a more practical and observational viewpoint. Together, their contributions spurred ongoing theoretical development and empirical investigation of overvalued ideation. These foundational perspectives provide the conceptual groundwork for contemporary models, which increasingly seek to integrate historical insights within multidimensional and process-based frameworks.

## 4. Contemporary Models of Overvalued Ideas

Building on the conceptual groundwork laid by Wernicke, Kraepelin, Jaspers, and Lewis, contemporary researchers have sought to refine and reframe overvalued ideas. Conflicting models and emerging empirical findings have complicated and refined the concept, contributing to the relative underuse of the term “OVI” compared with obsessions and delusions in research and practice. Although the term ‘OVI’ has been relatively underutilized in recent years, some researchers have attempted to synthesize existing models. Despite continued conceptual fragmentation, contemporary approaches increasingly converge toward a multidimensional understanding of overvalued ideation. To develop a comprehensive framework, it is necessary to examine how the construct has evolved across modern theoretical and empirical approaches.

### 4.1. Cognitive-Behavioral and Dimensional Perspectives

One dominant framework for conceptualizing OVIs is the insight-based continuum model. Kozak and Foa (1994) were among the first to situate overvalued ideas within a cognitive-behavioral framework, particularly in relation to obsessive–compulsive disorder (Kristinsdottir et al., 2025). The relationship between overvalued ideation and OCD is central to contemporary conceptualizations. While obsessions are typically ego-dystonic, intrusive, and recognized as irrational, overvalued ideas reflect a shift toward ego-syntonic, value-consistent beliefs that are experienced as justified and meaningful. In some individuals with OCD, beliefs may transition from intrusive doubts to more fixed, conviction-driven appraisals, increasing rigidity and resistance to change. Kozak and Foa (1994) observed that some individuals with OCD do not experience their beliefs as irrational or intrusive, but rather as plausible, value-laden convictions that motivate and sustain compulsive behavior (Wolf et al., 2023). They proposed that OVIs occupy a position along a continuum of insight, extending from obsessional doubt toward delusional conviction. They identified key features of OVIs—including high conviction, cognitive rigidity, and resistance to change—that they argued may undermine the effectiveness of exposure-based interventions. Importantly, they distinguished OVIs from delusions by emphasizing their psychological comprehensibility and affective investment, aligning their account with Wernicke’s original formulation.

### 4.2. Clinical Relevance and the Overvalued Ideas Scale

Building upon Kozak and Foa (1994), Neziroglu et al. (1999, 2001) advanced the study of overvalued ideas by developing the Overvalued Ideas Scale (OVIS), a clinician-rated instrument designed to assess belief dominance, emotional investment, and behavioral impact, with subsequent research continuing to support its role in assessing insight, belief strength, and resistance to modification (Neziroglu et al., 1999, 2001; Wolf et al., 2023). Applied primarily to OCD and body dysmorphic disorder (BDD), OVIS scores were shown to predict poorer treatment outcomes, suggesting that overvalued ideas function as a clinically relevant maintenance process. A similar process is observed in BDD, where beliefs about perceived appearance defects often function as overvalued ideas. Unlike intrusive obsessions, these beliefs are typically experienced as accurate and self-defining, reflecting strong value-based investment in appearance and identity. Neziroglu et al. (1999, 2001) emphasized that OVIs are rigid, value-driven belief systems rather than transient cognitive distortions and that they bridge cognitive and affective domains. Their findings support the view that overvalued ideation operates across diagnostic categories, consistent with a transdiagnostic, process-level mechanism. 

### 4.3. Eisen and Colleagues and the Brown Assessment of Beliefs Scale

Prior to the work by Neziroglu et al. (1999, 2001), Eisen et al. (1998) contributed to the dimensional understanding of belief pathology through the development of the Brown Assessment of Beliefs Scale (BABS), which assesses conviction, insight, and resistance across diagnostic groups. Their work demonstrated that belief certainty varies systematically across disorders, challenging categorical distinctions between obsessions, overvalued ideas, and delusions. Although not specific to OVIs, the BABS provided empirical support for conceptualizing overvalued ideas along a continuum of belief conviction rather than as a discrete diagnostic entity.

### 4.4. David Veale’s Value-Based Model

Moving beyond insight and dimensional models, value-based frameworks shift the focus toward the role of identity, meaning, and emotional reinforcement in sustaining overvalued ideas. Veale (2002) provided a comprehensive conceptual analysis of overvalued ideas. He synthesized cognitive-behavioral and phenomenological literature, proposing that overvalued ideas (OVIs) are beliefs rooted in idealized values with which individuals identify so intensely that these values come to encompass their sense of self. Veale emphasized that, unlike simple obsessions or delusions, OVIs are ego-syntonic, value-driven, and emotionally reinforced through behavioral actions, social validation, and emotional processes, becoming deeply integrated into one’s identity (Chavez-Baldini et al., 2024). He also noted that OVIs are often more resistant to treatment than clear obsessions, recommending therapeutic strategies that focus on value reappraisal and reframing, behavioral dominance, and emotional reinforcement, rather than merely challenging the content of beliefs.

**Figure 2:**
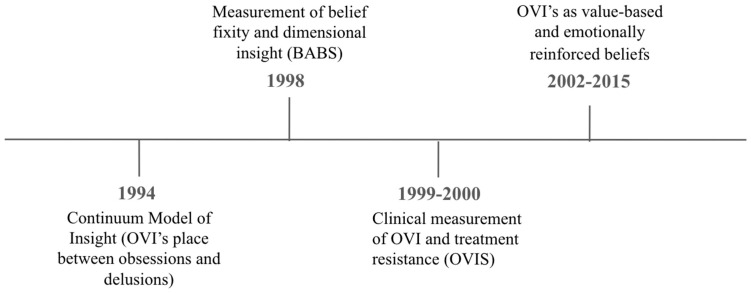
Evolution of contemporary models of overvalued ideas, from insight-based continuum models to dimensional belief assessment, clinical measurement, and value-based conceptualizations (Eisen et al., 1998; Kozak & Foa, 1994; Neziroglu et al., 2001; Veale, 2002). Note. Figure created by the authors.

As shown in Figure 2, These contemporary contributions highlight several key insights. First, overvalued ideas function as emotionally charged, value-laden beliefs, rather than solely as cognitive distortions. Second, they exist along a spectrum from obsession to delusion rather than fitting into distinct categories. Third, these ideas appear to operate through a mechanistic process across various disorders, such as OCD and BDD, rather than being confined to a single diagnosis. Finally, these findings emphasize the need for a multidimensional definition of overvalued ideas that encompasses conviction, affective influences, reinforcement patterns, subsequent behavioral actions, insight, and ego-syntonicity, rather than relying solely on insight, belief strength, or content-based labels. This process-level framing underlies the remaining sections of the review.

#### Obsessive–Compulsive Personality Disorder and Overvalued Ideation

The conceptual features articulated in Veale’s value-based model and Lewis’ applied model also invite consideration of obsessive–compulsive personality disorder (OCPD), which shares several formal characteristics with overvalued ideation. OCPD is defined by pervasive patterns of rigidity, perfectionism, moral inflexibility, and excessive devotion to productivity or control, all of which are experienced as ego-syntonic and justified by personal values (American Psychiatric Association, 2022). While there is notable overlap between OCD and OCPD, these are distinct disorders with different diagnostic criteria, courses, and treatment methods. OCPD is categorized as a cluster C personality disorder and is, according to Rizvi and Torrico (2023), “characterized by a general pattern of concern with orderliness, perfectionism, and control”. Unlike OCD, in which intrusive thoughts are typically ego-dystonic and resisted, individuals with OCPD rarely experience their beliefs as unwelcome or irrational. Instead, distress more often arises from interpersonal conflict, functional impairment, or external consequences rather than from the beliefs themselves. This phenomenological profile parallels structural aspects of overvalued ideas, particularly the alignment between belief, identity, and perceived moral or personal correctness.

However, OCPD should not be equated with an overvalued idea itself but rather understood as a personality organization that may scaffold or stabilize overvalued ideation over time. Whereas overvalued ideas are belief-centered phenomena characterized by emotional amplification and behavioral dominance, OCPD reflects a broader, trait-level configuration in which rigidity and value fusion are generalized across domains (P. J. McKenna, 1984; Pinto et al., 2022). Within such a structure, specific beliefs may acquire disproportionate importance and resistance to change, functioning as overvalued ideas embedded within a stable personality framework. This distinction preserves conceptual clarity while illustrating how overvalued ideation can be episodic, chronic, or personality-stabilized depending on the broader psychological context.

Considering OCPD in relation to overvalued ideation further supports the argument that OVIs represent a transdiagnostic mechanism rather than a disorder-specific feature. The presence of OVI-like belief dominance across obsessive–compulsive, body image, eating, and personality-related conditions suggests a shared cognitive–affective architecture grounded in value alignment, affective reinforcement, and behavioral rigidity. While neurobiological findings remain limited regarding OCPD, the prominence of self-referential valuation, narrative coherence, and inflexibility in both OCPD and OVIs is consistent with cognitive–behavioral models emphasizing value-driven belief salience and identity-level rigidity rather than focal pathology (Barton et al., 2022; Veale, 2002). Including OCPD within this framework thus broadens the scope of overvalued ideation, demonstrating its relevance across both symptom-based and trait-based forms of psychopathology and reinforcing its utility as a process-level construct.

## 5. Discussion

Despite over a century of theoretical discussion and historical richness, the term overvalued idea remains inconsistently defined and underutilized due to conceptual ambiguity. Since Wernicke’s original description, attempts to define OVIs have been complicated by the clash of intersecting frameworks rather than by an integration of historical foundations and modern perspectives—categorical, dimensional, or cognitive-behavioral. To advance theoretical clarity, it is essential to first disentangle common misconceptions about OVIs and then identify the features consistently supported by both historical and contemporary literature. Finally, it is important to strike a balance between theoretical completeness and practical utility. This final section integrates the preceding historical and contemporary analyses to clarify misconceptions, define core features, situate overvalued ideation within a multidimensional, transdiagnostic framework, and ties this analysis back to Wernicke’s original position.

### 5.1. Common Assumptions and Misconceptions of Overvalued Ideas

#### 5.1.1. One-Dimensional Continuum of Insight

One of the most prominent assumptions made by researchers, psychologists, and organizations is that an overvalued idea exists on a one-dimensional continuum of insight, positioned between obsessions and delusions. As Veale (2002, p. 319) states, “For authors in the USA an over-valued idea has become shorthand for ‘poor insight’ in the middle of a continuum of obsessional doubts to delusional certainty”. This perspective is not only ambiguous but also oversimplifies the complex nature of overvalued ideas. While it may be useful in this manner, it does not provide the full and deeply nuanced picture. Such conceptualizations may rely too heavily on insight as the primary indicator of overvalued ideation. Furthermore, the model fails to specify the mechanisms that drive movement along this continuum and does not consider other potential dimensional characteristics. While limited insight into an individual’s psyche or disordered thinking is a significant aspect of overvalued ideation, it does not provide a complete understanding.

#### 5.1.2. Equating Overvalued Ideas with Delusions

The conceptual complexity surrounding OVIs has led researchers and clinicians to rely on more mainstream and widely understood concepts and definitions. As a result, OVIs are easily misconstrued as “mild delusions,” an unintentional and misguided label that limits its utility in research and clinical contexts (Barton et al., 2022; Mullen & Linscott, 2010; Veale, 2002). Construing OVIs as “mild” delusions underemphasizes the aspect of understandability emphasized by Wernicke and potentially overlooks the root causes of certain maladaptive thought patterns that may contribute to psychological disorders. This perspective also overlooks important distinctions related to their developmental and social characteristics. Recent work has emphasized that OVIs are not typically idiosyncratic like delusions and, instead, often emerge from socially or culturally shared beliefs or belief systems which become increasingly rigid and dominant (Crisan et al., 2022; Mullen & Linscott, 2010; Rahman et al., 2020). This label frames an overvalued idea in terms of its relationship with an individual’s personality, values, and lived experiences. OVIs represent belief systems that are distinct from the unintelligible belief structures characteristic of psychosis.

#### 5.1.3. Equating Overvalued Ideas with Obsessions

Obsessions, often positioned at the opposite end of the insight continuum, are a concept related to but often conflated with overvalued ideas. This common misunderstanding is reflected in several DSM and ICD formulations. Obsessions, such as those experienced with OCD, are characterized by ego-dystonic intrusions. In contrast to OVIs, obsessions are unwanted, distressing, and inconsistent with one’s self-concept, identity, and personality. This distinction is critical as it delineates two separate motivational systems: one driven by anxiety and avoidance, and the other by value-based behavior and reinforcement. In a clinical context, failing to properly identify the causes of these maladaptive thought patterns may limit the effectiveness of treatments. Kozak and Foa (1994) and Eisen et al. (1998) demonstrated that patients with OCD or BDD often experience beliefs and ideas that fall between these categories, and that the cognitive–affective mechanisms underlying overvalued ideas remain distinct from the intrusive and unwanted nature of obsessions, with recent work continuing to emphasize the nuanced distinctions between delusions, overvalued ideas, and obsessions in clinical assessment (Serdenes et al., 2023). Related work on binge eating further underscores the importance of distinguishing stable belief dominance from transient cognitive states. Heatherton and Baumeister’s (1991) escape-from-self-awareness model conceptualizes binge eating not as the expression of a fixed or ego-syntonic belief, but as a motivated shift into a narrowed cognitive state in which reflective self-awareness and critical evaluation are temporarily reduced (Lin et al., 2025). In this deconstructed state, individuals may endorse irrational or poorly evaluated thoughts, not because these beliefs are central to identity or values, but because meaningful self-referential processing is actively avoided. From the present perspective, overvalued ideation may operate upstream by intensifying self-discrepancy and affective salience, thereby increasing the aversiveness of self-awareness. Binge eating, in this context, can be understood as a behavioral consequence of escaping an overvalued self-concept rather than as the direct enactment of an overvalued idea itself.

#### 5.1.4. Measuring Overvalued Ideas Solely by Conviction

A further conceptual pitfall is the tendency to measure or define OVIs solely by the strength of belief or conviction. This approach overlooks the multidimensional interplay of cognitive, affective, and value-based processes that contribute to such fixation. Veale (2002) and Neziroglu et al. (2001) argue that the defining feature of an OVI is not merely its strength, but its functional dominance in an individual’s mental life (Kristinsdottir et al., 2025). They emphasize the extent to which OVIs influence behavior, motivate action, and resist disconfirmation because of their emotional salience. The pathological threshold is reached when emotional reinforcement sustains the idea despite overwhelming counterevidence or negative consequences.

At the core of the OVI is emotional overvaluation, which transforms a normal belief into one infused with excessive emotional significance. Wernicke (1900) identified this mechanism in affective processes that amplify the importance of an idea within associative networks. Modern theorists echo this view, describing OVIs as value-laden beliefs maintained by affective reinforcement rather than by evidence, often leading to behavioral outcomes (Veale, 2002; Kristinsdottir et al., 2025; Overholser, 2018). Emotional salience sustains its dominance, ensuring its persistence even in the face of contradictory information. Clarifying these misconceptions allows for a more precise articulation of the core features of overvalued ideation.

### 5.2. Core Features of Overvalued Ideas

#### 5.2.1. Ego-Syntonicity and Value-Based Meaningfulness

The ego-syntonic nature of overvalued ideas (OVIs) is one factor that sets them apart from both obsessions and delusions, as shown in Table 1. These beliefs align with the individual’s self-concept, values, and moral framework, providing a sense of purpose or coherence. Jaspers (1913/1959, 1913/1963) referred to this phenomenon as understandability—the idea that it can be traced back to personal motivations, values, or experiences (Wendler & Fuchs, 2023). Because the individual perceives the idea as rational, they rarely seek to resist it, complicating intervention efforts. Veale (2002) emphasized the significance of value alignment and how overvalued ideas emerge from idealized and inflated values (Barton et al., 2022).

#### 5.2.2. Cognitive Rigidity and Resistance to Change

Although not entirely fixed or bizarre, OVIs exhibit a characteristic resistance to modification or counterevidence. Individuals may acknowledge alternative perspectives but interpret them through the lens of their OVI, continuing to justify their own stance. In some cases, alternate perspectives may reinforce their resistance to change. Cognitive rigidity serves as a defensive mechanism, maintaining emotional equilibrium and a sense of control. This feature parallels mechanisms described in cognitive-behavioral models of obsessional and body image disorders (Kendler, 2018; Neziroglu et al., 2001).

#### 5.2.3. Emotional Amplification and Cognitive–Affective Reinforcement

Multiple frameworks converge on exaggerated emotionality as a key marker in the development and maintenance of OVIs. Wernicke’s early descriptions of associative networks have been interpreted as emphasizing that emotionality can cause an otherwise ordinary idea to acquire disproportionate salience and dominate a person’s mental life and behavior, without necessarily becoming psychotic in form (Rahman et al., 2020). Veale (2002) and Kristinsdottir et al. (2025) similarly highlight emotional reinforcement as central to OVIs, particularly when the belief becomes integrated with identity and values, such that it feels self-defining and therefore requires defense rather than revision, even in the face of contradictory evidence.

OVIs can be conceptualized as forming through a cognitive–affective reinforcement loop: (a) an idea becomes linked to a core value, identity, or perceived threat; (b) affective arousal (e.g., anxiety, shame, fear, anger) increases the subjective importance of the idea; (c) behaviors aimed at reducing distress (e.g., reassurance-seeking, checking, appearance-fixing rituals, cleaning, dietary restriction, and avoidance) provide short-term emotional relief or validation; and (d) this relief acts as reinforcement, strengthening conviction and rigidity, and narrowing attention around the belief. The exaggerated emotionality associated with the belief not only further reinforces it but may also drive the belief into pathological dominance, contributing to the development of various disorders. It’s also plausible that emotional dysregulation can serve as a marker for a pathological-level salience (Dork et al., 2024). While more research is needed to confirm a connection, preexisting emotional dysregulation may act as a precursor or contributing factor to overvalued ideation. When individuals have limited access to emotion-regulation strategies, emotionally charged appraisals may become more persistent and compelling, increasing the reliance on repetitive, compulsion-like behaviors associated with the belief.

#### 5.2.4. Behavioral Dominance and Functional Impairment

A defining feature of OVIs is their strong influence on behavior. An OVI can eventually guide behavior disproportionately more than other core beliefs or values. It may become a guiding principle that shapes daily decisions, rituals, and avoidance behaviors. Heatherton and Baumeister (1991) note that reductions in higher-order self-regulation can narrow attention to immediate emotional relief, facilitating repetitive behaviors that reinforce and sustain dominant belief systems (Lin et al., 2025). In conditions such as body dysmorphic disorder, binge-eating or anorexia nervosa, overvalued beliefs about appearance or weight can lead to pervasive behavioral patterns that are difficult to disrupt (Eisen et al., 1998; Heatherton & Baumeister, 1991; Lin et al., 2025). Consequently, an OVI can create a self-reinforcing cycle in which actions reinforce convictions, which in turn justify further actions. As this pattern strengthens, the individual may begin to experience difficulties concentrating, performing at work or school, maintaining a healthy physical and social lifestyle, and experience declines in overall mental well-being.

#### 5.2.5. Limited Insight

While early models positioned OVIs as fixed points on a linear continuum between normal thought and delusion, modern perspectives recognize that insight fluctuates both contextually and over time. Veale (2002) and Pierre (2019) suggest that insight operates within a multidimensional system influenced by emotional regulation, cognitive bias, and social reinforcement; consistent with this view, longitudinal findings indicate that insight is dynamic and associated with symptom severity and functioning (Wolf et al., 2023), while recent clinical work continues to emphasize the nuanced distinctions between delusions, overvalued ideas, and obsessions within this dimensional framework (Serdenes et al., 2023). Although previous definitions of OVIs may have overemphasized the influence of insight, it remains an important aspect of the concept. OVIs are characterized by some level of diminished insight into one’s disorder or rigid thinking. More research is needed to determine where on the insight continuum this point lies, how much variability can be observed, how insight is lost or gained, what determines whether the introduction of counterevidence raises or lowers that level, and how emotional salience or associated affect drives this movement. It can reasonably be assumed that a combination of high affect and moderate to low insight is most likely to foster OVIs and lead to greater resistance to new information or conflicting evidence.

#### 5.2.6. A Transdiagnostic Mechanism

The salience of ideas resembling overvalued ideation suggests that OVIs follow a transdiagnostic pattern. Although more research is needed to fully understand the nature and function of OVIs, they appear to represent a transdiagnostic cognitive function, an ingrained pattern of thought, or an individual tendency rather than a singular diagnosis. This tendency is reinforced over time. Based on this assumption, the process of overvalued ideation is not inherently pathological; however, if it is excessively reinforced and emotionally attached to a specific idea, it can cross the threshold into pathology, becoming pervasive and damaging to an individual’s well-being. Thus, OVIs can be understood as the result of a cognitive mechanism or process that distorts certain ideas with pathological intensity and manifests across various diagnoses. While this understanding is theoretically grounded, it requires further research. Exploring this component may be crucial for enhancing treatment methods for disorders associated with OVIs.

#### 5.2.7. Proposed Operational Definition of Overvalued Ideas

Drawing from the synthesis of historical and contemporary literature, the following definition is proposed: An overvalued idea is an emotionally amplified, rigid, ego-syntonic belief that originates from normal cognition but becomes disproportionately significant, dominating cognition and behavior through affective and behavioral reinforcement. Such ideas contribute to the pathological distress accompanying various psychological disorders.

### 5.3. Was Wernicke Right? Reframing an Old Debate

The historical debate surrounding the nature of overvalued ideas has often been framed as a dichotomy between psychological intelligibility and biological pathology. Karl Wernicke’s original formulation emphasized overvalued ideas as psychologically understandable phenomena arising from normal associative processes that become pathologically amplified through affective reinforcement. In contrast, Kraepelin’s nosological framework subsumed similar phenomena under biological disease progression, implicitly treating such beliefs as early or attenuated expressions of psychosis. While these positions have frequently been cast as mutually exclusive, this opposition is overstated.

Wernicke’s central position, that overvalued ideas are non-delusional, ego-syntonic beliefs comprehensible within an individual’s personality, values, and experience, is supported by contemporary cognitive-behavioral and phenomenological models (Barton et al., 2022; Crisan et al., 2022; Ritunnano et al., 2023). These frameworks demonstrate that overvalued ideas are sustained through emotional salience, reinforcement contingencies, and value-based meaning rather than through fundamental loss of reality testing. Modern network-based perspectives further reinforce Wernicke’s insight by conceptualizing these beliefs as arising from dysregulated associative dominance rather than focal neurological lesions. Consistent with Hebbian learning principles, repeated co-activation of associative networks increases the likelihood of future activation, strengthening the salience and dominance of the belief (Halvagal & Zenke, 2023). Over time, these reinforced pathways may contribute to a generalized vulnerability toward overvalued ideation or consolidate into stable associative networks corresponding to specific overvalued ideas. Importantly, the psychological comprehensibility aspect of overvalued ideation does not trivialize the clinical significance of overvalued ideas; rather, it helps explain their persistence, rigidity, and resistance to change.

At the same time, Kraepelin’s observations regarding prognosis and chronicity cannot be dismissed (Heckers, 2020; Insel et al., 2022). Overvalued ideas frequently signal poorer treatment outcomes across multiple disorders, and pharmacological interventions may attenuate the emotional intensity or behavioral dominance associated with such beliefs. However, responsiveness to biological intervention doesn’t narrowly imply a biological etiology nor exclude the modulation of affective and salience-related processes. Kraepelin’s contribution is therefore best understood as descriptively accurate with respect to illness course, but etiologically incomplete. His framework captured observable regularities in illness course but was limited in its explanatory account of belief formation.

Karl Jaspers’ phenomenological approach, of which elements are still applied in modern research, provided a critical bridge by distinguishing overvalued ideas from delusions based on psychological understandability rather than conviction alone (Jaspers, 1913/1963; Ritunnano et al., 2023). His emphasis on meaning, motivation, and experiential continuity preserved both scientific rigor and phenomenological clarity, establishing overvalued ideas as a distinct form of belief rather than an attenuated psychosis.

Aubrey Lewis extended this distinction into clinical practice, describing overvalued ideas as emotionally charged, plausible, and behaviorally directive beliefs characterized by partial insight and resistance to modification. Contemporary research continues to reflect these features, with recent studies emphasizing the role of emotionally salient, strongly held beliefs that are resistant to modification and associated with varying levels of insight across conditions such as obsessive–compulsive and related disorders, supporting the continued relevance of Lewis’s characterization of overvalued ideas (Barton et al., 2022). By emphasizing their disproportionate influence on behavior, Lewis demonstrated how psychologically intelligible beliefs can still exert pathological control, reinforcing both Wernicke’s and Kraepelin’s core observations within a practical clinical framework.

### 5.4. Limitations

The current review explores historical and contemporary literature and frameworks pertaining to overvalued ideas and highlights their academic and clinical relevance. While intended, this review is limited by its narrative and conceptual approach, as it does not involve systematic search procedures or quantitative synthesis of findings (M. N. Ahmad, 2025). As a result, the selection and interpretation of sources may be subject to bias, and the conclusions drawn may reflect theoretical positions rather than empirical validation (M. N. Ahmad, 2025).

As discussed throughout this paper, the existing empirical literature is currently relatively scarce. Despite being a driving factor in the development of this conceptual review, it also acts as a limitation. This constrains the ability to draw definitive conclusions regarding OVIs, their structure, and clinical application (M. N. Ahmad, 2025). While this review aims to clarify these inconsistencies, the proposed multidimensional framework requires further empirical testing and validation across clinical populations. Consequently, the need for future empirical research remains central.

## 6. Future Research and Clinical Applications

This review presents overvalued ideation as a multidimensional, transdiagnostic mechanism rather than a disorder-specific feature or attenuated form of delusion. Systematic empirical work is now needed to test, refine, and operationalize this framework. The following sections also outline key clinical implications intended to improve diagnostics and treatment efficacy.

### 6.1. Future Research Directions

The current review underscores substantial opportunities for advancing empirical research on overvalued ideas. While the construct has received sustained theoretical attention, the possibilities and directions for future OVI research are vast, as it has not previously been systematically operationalized or empirically evaluated across different populations, leaving important questions about its measurement, mechanisms, and clinical significance unanswered.

#### 6.1.1. Confirming Key Features and Model Components

We propose a multidimensional transdiagnostic model of overvalued ideas; however, empirical research is needed to confirm its core features identified in prior sections (e.g., ego-syntonicity, affective reinforcement, behavioral dominance). At present, many of these features are grounded primarily on theoretical foundations, with limited direct empirical testing.

Such validation could be achieved using the existing OVIS or a future validated diagnostic measure or descriptive tool suitable for broader use. Further investigation is needed to understand how these features change over time and interact with one another. Future research should examine how these components emerge, interact, and change over time, including whether specific features precede OVI development, vary across disorders, or cluster around identity-relevant values. Researchers should determine whether effective treatment requires targeting a single dominant (e.g., rigidity/fixity of thinking, values, associated affect, insight, etc.) or multiple components simultaneously.

Multivariate and latent-variable approaches may be useful in displaying how these dimensions reliably cohere into a distinct construct across diagnostic categories. Additionally, confirmatory factor analysis, network modeling, and bifactor approaches may be useful in determining whether overvalued ideation constitutes a unified process or a constellation of partially independent mechanisms. Establishing this structure is a necessary step toward improving measurement, refining theoretical models, and identifying meaningful intervention targets.

#### 6.1.2. Measurement and Psychometric Tool Development

The only validated tool for measuring overvalued ideation currently available to researchers and clinicians is the OVIS. However, it is only validated as a clinically administered tool and is unsuitable for self-report use. This significantly constrains the scope of empirical research on OVIs. Factor-analytic methods should be employed to test competing structural models and to establish measurement invariance across clinical and non-clinical samples. A valid self-report measure of OVIs that captures factors introduced to the existing literature since the release of the original OVIS would greatly expand research possibilities and ultimately enhance our understanding of OVIs across populations, as well as facilitate the evaluation of treatment methods and effectiveness.

#### 6.1.3. Mechanisms of Belief Amplification and Transition

A recurrent theme in the literature surrounding overvalued ideas (OVIs) is the dimension of insight. Future research should investigate which mechanisms determine how a belief may shift along this continuum. Longitudinal and experimental designs would be useful in identifying factors that intensify emotional salience, increase rigidity, or erode insight, thereby transforming a relatively circumscribed overvalued idea into a more severe or treatment-resistant belief. Particular attention should be paid to affective reinforcement processes, emotion-regulation capacity, and social validation. This distinction is particularly important for understanding the heterogeneity in clinical outcomes and for identifying targets for early intervention. It should further be studied whether multiple belief or idea types can occur simultaneously and whether OVIs, delusions, and obsessions may co-occur and interact dynamically, potentially amplifying symptom severity and influencing treatment response.

#### 6.1.4. Population-Based Variance

Further research is needed to explore the differences in content and thematic focus of OVIs between clinical and non-clinical populations. Many socially sanctioned belief systems—such as those related to morality, health, achievement, identity, or ideology—may exhibit features of overvalued ideation without crossing into disorder. Comparative studies across different clinical and non-clinical populations may help delineate distinctions between normative exaggerated beliefs and pathological dominance, refining the thresholds for clinical significance. This research is vital for validating OVIs as a multidimensional construct and for understanding the mechanisms by which ideas become pathological.

### 6.2. Clinical Implications

This review conceptualizes overvalued ideation as a multidimensional, transdiagnostic process with significant implications for clinical assessment, diagnosis, and intervention. Instead of confining pathological beliefs to specific diagnostic categories or content, this framework urges clinicians to assess how these beliefs operate within an individual’s emotional, cognitive, and behavioral systems, and consider how pathological ideas form, operate, and affect treatment outcomes.

#### 6.2.1. Assessment and Diagnostics

A mechanistic understanding of overvalued ideation highlights the importance of assessing belief characteristics beyond conviction or factual accuracy. A comprehensive and operationalized model will help clinicians understand how ideas become exaggerated to a pathological degree, and how they differ from other types of ideas (i.e., obsessions and delusions). Distinguishing factors should include emotional investment, value alignment, behavioral dominance, insight level, rigidity, ego-syntonicity, cultural context, etc. Early and accurate identification of overvalued ideas among clients is important for diagnostics that guide treatment methods.

#### 6.2.2. Treatment Planning and Resistance

A more thorough and widespread understanding and assessment of OVIs across clinical settings may help improve treatment outcomes for multiple disorders. Research has shown that people experiencing overvalued ideas are significantly more resistant to treatment, even compared with obsessions and delusions (Veale, 2002). This may reflect a need for an OVI-direct intervention extending beyond standard cognitive-behavioral techniques. Therapeutic approaches may need to shift away from challenging belief content alone and toward modifying the mechanisms that sustain belief dominance. This would involve process-based therapies, which emphasize emotional regulation, values clarification, and behavioral experimentation rather than direct disputation of belief accuracy. Clinically, this distinction suggests that behaviors such as binge eating may reflect attempts to escape aversive self-awareness intensified by overvalued beliefs, rather than the direct expression of those beliefs themselves. Importantly, targeting overvalued ideation does not require eliminating deeply held values, but rather loosening their absolute authority over behavior and self-concept.

Importantly, distinguishing OVIs from delusions requires careful evaluations of their psychological understandability, process, and intensity. Making this distinction can help reduce premature psychotic labeling and avoid unnecessary escalation to antipsychotic interventions when insight is partially preserved. This may help ensure that treatment methods are neither inadequate and symptom-targeting nor overly reactive and imposing, consistent with the principle of conservative treatment and least harm. A multidimensional model of overvalued ideation supports more personalized treatment planning, especially when patients differ in which components are most salient. For some, affective regulation targets may be necessary for cognitive flexibility, while in other cases, behavioral modification methods may be needed to weaken reinforcement loops. Integrating this concept into education and clinical practice may refine diagnostic and treatment methods and enhance overall outcomes.

## 7. Conclusions

Viewed through this integrative lens, the enduring relevance of the overvalued idea lies not in adjudicating a historical dispute, but in clarifying a shared psychological process that operates across diagnostic boundaries. Overvalued ideation is best conceptualized as a transdiagnostic process in which emotionally amplified, ego-syntonic beliefs exert disproportionate influence over cognition and behavior, reflecting a distinct multidimensional configuration that differentiates it from ego-dystonic obsessions and delusional beliefs. This process may manifest across obsessive–compulsive disorder, body dysmorphic disorder, eating disorders, and related conditions.

Understanding overvalued ideation in this way allows clinicians and researchers to move beyond static diagnostic classifications toward process-based assessment and intervention targeting the mechanisms that sustain belief dominance. In doing so, the construct retains its original phenomenological meaning while gaining renewed clinical and empirical utility, positioning overvalued ideation as a key link between belief, emotion, and behavior across diverse forms of psychopathology and informing efforts to improve treatment outcomes.

## Figures and Tables

**Table 1:** Distinguishing OVIs from delusions and obsessions based on typical patterns.

Feature	Obsession	OVI	Delusion
Insight	Moderate-high	Partial/Poor	Absent
Conviction	Low	High	Very high/Absolute
Ego-syntonicity	Ego-dystonic/Intrusive	Ego-syntonicity	Mostly ego-syntonic
Rigidity	Low-moderate. Appraisals are more easily shifted	High. Often defended and amplified	Very high/Absolute
Affective Investment	Marked by anxiety/distress	High affective salience/Emotionally driven	Variable emotional distress
Behavioral Dominance	Compulsions/avoidance behaviors	Disproportionate influence on behavior	Behaviors may be organized around delusions

## Data Availability

The original contributions presented in this study are included in the article. Further inquiries can be directed to the corresponding authors.
